# Multiple SARS-CoV-2 Introductions Shaped the Early Outbreak in Central Eastern Europe: Comparing Hungarian Data to a Worldwide Sequence Data-Matrix

**DOI:** 10.3390/v12121401

**Published:** 2020-12-06

**Authors:** Gábor Kemenesi, Safia Zeghbib, Balázs A Somogyi, Gábor Endre Tóth, Krisztián Bányai, Norbert Solymosi, Peter M Szabo, István Szabó, Ádám Bálint, Péter Urbán, Róbert Herczeg, Attila Gyenesei, Ágnes Nagy, Csaba István Pereszlényi, Gergely Csaba Babinszky, Gábor Dudás, Gabriella Terhes, Viktor Zöldi, Róbert Lovas, Szabolcs Tenczer, László Kornya, Ferenc Jakab

**Affiliations:** 1 National Laboratory of Virology, Szentágothai Research Centre, University of Pécs, 7624 Pécs, Hungary; zeghbib.safia@gmail.com (S.Z.); somogyi.balazs@pte.hu (B.A.S.); toth.gabor.endre@gmail.com (G.E.T.); 2 Institute of Biology, Faculty of Sciences, University of Pécs, 7624 Pécs, Hungary; 3 Institute for Veterinary Medical Research, Centre for Agricultural Research, 1093 Budapest, Hungary; bkrota@hotmail.com; 4 Centre for Bioinformatics, University of Veterinary Medicine Budapest, 1078 Budapest, Hungary; solymosi.norbert@gmail.com; 5 Translational Discovery, Stromal Biology, Bristol-Myers Squibb, Princeton, NJ 08648, USA; dr.szabo.peter@gmail.com; 6 Veterinary Diagnostic Directorate, National Food Safety Office, 1143 Budapest, Hungary; iszabodr@t-online.hu (I.S.); BalintAd@nebih.gov.hu (Á.B.); 7 Bioinformatics Research Group, Genomics and Bioinformatics Core Facility, Szentágothai Research Centre, University of Pécs, 7624 Pécs, Hungary; urpe89@gmail.com (P.U.); herczeg.robert@pte.hu (R.H.); gyenesei.attila@pte.hu (A.G.); 8 Clinical Research Centre, Medical University of Bialystok, 15-089 Bialystok, Poland; 9 Medical Centre, Hungarian Defense Forces, 1114 Budapest, Hungary; balazs.nagy.agnes@hmei.hu (Á.N.); pereszlenyi.csaba@hm.gov.hu (C.I.P.); babinszky.gergely@hm.gov.hu (G.C.B.); gabor.dudas@hm.gov.hu (G.D.); 10 Institute of Clinical Microbiology, Faculty of Medicine, University of Szeged, 6720 Szeged, Hungary; terhes.gabriella@med.u-szeged.hu; 11 Independent Researcher, 1301 Vantaa, Finland; viktor.zoldi@gmail.com; 12 Institute for Computer Science and Control (SZTAKI), Eötvös Loránd Research Network, 1111 Budapest, Hungary; robert.lovas@sztaki.hu (R.L.); tenczer.szabolcs@sztaki.hu (S.T.); 13 Central Hospital of Southern Pest—National Institute of Hematolgy and Infectious Diseases, 1476 Budapest, Hungary; kornya@kornya.com

**Keywords:** phylodynamics, network analysis, SARS-CoV-2, human coronavirus, pandemic, outbreak

## Abstract

Severe Acute Respiratory Syndrome Coronavirus 2 is the third highly pathogenic human coronavirus in history. Since the emergence in Hubei province, China, during late 2019, the situation evolved to pandemic level. Following China, Europe was the second epicenter of the pandemic. To better comprehend the detailed founder mechanisms of the epidemic evolution in Central-Eastern Europe, particularly in Hungary, we determined the full-length SARS-CoV-2 genomes from 32 clinical samples collected from laboratory confirmed COVID-19 patients over the first month of disease in Hungary. We applied a haplotype network analysis on all available complete genomic sequences of SARS-CoV-2 from GISAID database as of 21 April 2020. We performed additional phylogenetic and phylogeographic analyses to achieve the recognition of multiple and parallel introductory events into our region. Here, we present a publicly available network imaging of the worldwide haplotype relations of SARS-CoV-2 sequences and conclude the founder mechanisms of the outbreak in Central-Eastern Europe.

## 1. Introduction

Following the 2002 SARS (Severe Acute Respiratory Syndrome) pandemic and the discovery of MERS (Middle Eastern Respiratory Syndrome) coronavirus in 2012, the third highly pathogenic human coronavirus in history emerged in Hubei province, China, during late 2019. The novel virus was subsequently named Severe Acute Respiratory Syndrome Coronavirus 2 (SARS-CoV-2) and the acute respiratory disease as coronavirus disease 19 (COVID-19) [1]. Currently, SARS-CoV-2 is responsible for the ongoing coronavirus pandemic spreading on all inhabited continents. As of 28 October 2020, the confirmed case numbers surpassed 44 million worldwide and the disease associated mortality rate exceeded 1.1 million [2].

At the onset of the second week of March, Europe became the next epicenter of the pandemic, following China, as reported by the World Health Organization [3]. By the end of April, more than one million laboratory confirmed cases were reported from all European countries [4]. The first two Hungarian cases were officially confirmed on March 4th, according to the data of ECDC Communicable Disease Threats Report [5]. Border closures and universal ban regarding public gatherings was announced on March 17.

To better comprehend the detailed founder mechanisms of the epidemic evolution in Central-Eastern Europe, particularly in Hungary, we determined the full-length SARS-CoV-2 genomes from 32 clinical samples collected from laboratory confirmed COVID-19 patients over the first month of disease in Hungary. Our virus sampling started from this date and spanned the first two weeks of country-wide mitigation regulations (17 March through 2 April 2020). In this study, we intend to understand the underlying mechanisms behind the successful mitigation of the first wave of epidemic in the Central-Eastern European region, focusing on Hungarian data. For this purpose we use various tools of genomic epidemiology, primarily the minimum spanning tree-based network analysis and as a secondary verifying method we performed general phylogenetic analysis as well

## 2. Materials and Methods

### 2.1. Sample Collection

Oro-pharyngeal swab samples were obtained from 32 patients during the period from March 17 to April 2. Within the frame of a country-wide collaboration network regarding SARS-CoV-2 research, nucleic-acid samples were received from University Hospitals at Szeged and Budapest and from the Hungarian Defense Forces, Military Medical Center. Ethical approval (approval date—31 January 2020) was obtained from the University of Pécs, Ethics Committee, under the registration number: 8218-PTE2020.

### 2.2. Direct Sequencing and Primary Data Analysis from Patient Samples

Nucleic acid samples were extracted directly from oro-pharyngeal swab samples using a Direct-zol™ RNA MiniPrep Plus extraction kit (Zymo Research, Irvine, CA, U.S.A.) and in full compliance to the manufacturers’ recommendations. Reverse transcription and multiplex PCR were performed on the basis of information provided by the Artic Network initiative [6]. Both the concentration and the quality of the PCR products were measured and checked using the Agilent 4200 TapeStation System (Agilent Technologies, Santa Clara, CA, U.S.A.) and ThermoFisher Scientific Qubit 3 Fluorometer (Thermo Fisher Scientific, Waltham, MA, United States). The 32 sequencing libraries were prepared using 98 overlapping amplicons covering the whole viral genome. The libraries were then quantitatively checked, barcoded, and sequenced on 5 flow cells using Oxford Nanopore MinION Flow Cells (R9.4.1) (Oxford Nanopore Technologies, Littlemore, Oxford OX4 4DQ, United Kingdom).

During primary data analysis, we used RAMPART to track the sequencing process in “real-time” in order to acquire instant information regarding the quality of samples and the coverage of the amplicons. Sequencing reads of samples with sufficient amplicon coverage were mapped and consensus sequences generated by the bioinformatics pipeline built within the Artic Network protocol.

### 2.3. Genome Data Analysis

SARS-CoV-2 genomes (*n* = 7864) were downloaded from GISAID database on 21 April 2020. Only complete (>29,000 base-pair length) and high quality (with <1% Ns, <0.05% unique amino acid mutations and no insertion/deletion unless verified by submitter) sequences were used for network construction. To quantify the sequence similarity, percent identity was calculated based on the BLAST [7] alignment for each paired sequence.

First, using the resulted similarity matrix, a fully connected, edge-weighted network was constructed, where each node represented a COVID sequence, while the edges represented their potential connections, and the edge weights (similarity values). Secondly, the edge weights were transformed (100-weight) in the full network to make high values low and low values high. Next, a minimum spanning tree (MST) was identified as described previously [8]. The path of MST is considered as the most probable chain of infection. Although it should be interpreted with caution, considering the underrepresentation of sequence data to the size of epidemic, this method is suitable to conclude the origin of an epidemic. In our case it means the verification of single or multiple introduction theories and associate Hungarian sequences with geographic regions of the epidemic. In a spanning tree, every node has only one or two connections. If multiple edges have the same minimum weight, the algorithm will randomly pick one and not select all links with the same values. To manage this issue, the graph with additional edges was modified by adding every edge for each node having an equal or higher weight than the edges in the initial MST to the corresponding node. All data analyses were performed using the R 3.6.2 on Linux [9], for network creation, and the Igraph package was applied [10].

In regard to the generation of time-scaled phylogenetic tree, 105 SARS-CoV-2 genomes were retrieved from GISAID [11] following a manual selection based on the network analysis. The sequences were aligned in MAFFT v.7 [12] with default parameters. Subsequently, both best-fitting substitution model and the maximum likelihood phylogenetic tree with ultra-bootstrapping were implemented in IQTREE webserver [13,14]. The resulting tree was subjugated to a root-to-tip regression analysis in TempEst [15] to assess the clock-likeness regarding the data. A positive correlation was observed between sampling time and root-to-tip genetic divergence (r = 70, R^2^ = 54) indicating the suitability of the dataset for molecular clock analysis using the Beast v1.10.4 package. The KHY+I substitution model with the uncorrelated lognormal relaxed clock, in addition to the coalescent exponential population growth model, were applied [16]. The MCMC chains were run for 200 million iterations and sampled every 10,000 cycles, or generations, with 10% discarded as burn in. We explored the effective sample sizes in Tracer (ESS > 200) [17]. Moreover, to explore the phylogeographic diffusion of SARS-COV-2 in continuous space, the lognormal relaxed random walk diffusion model and a lognormal uncorrelated relaxed clock model were implemented in the same package, were next employed. Thus, the maximum clade credibility tree was visualized in SpreaD3 [18].

Lineage assignment of the Hungarian sequences was performed using the PANGOLIN (Phylogenetic Assignment of Named Global Outbreak LINeages) software v1.0, which uses a recently published lineage nomenclature [19,20].

The datasets generated during and analysed during the current study are available in the NDEx-The Network Data Exchange repository, http://www.ndexbio.org/#/network/2c66e15b-8eeb-11ea-aaef-0ac135e8bacf (accessed on 10 November 2020).

## 3. Results and Discussion

**Figure 1:**
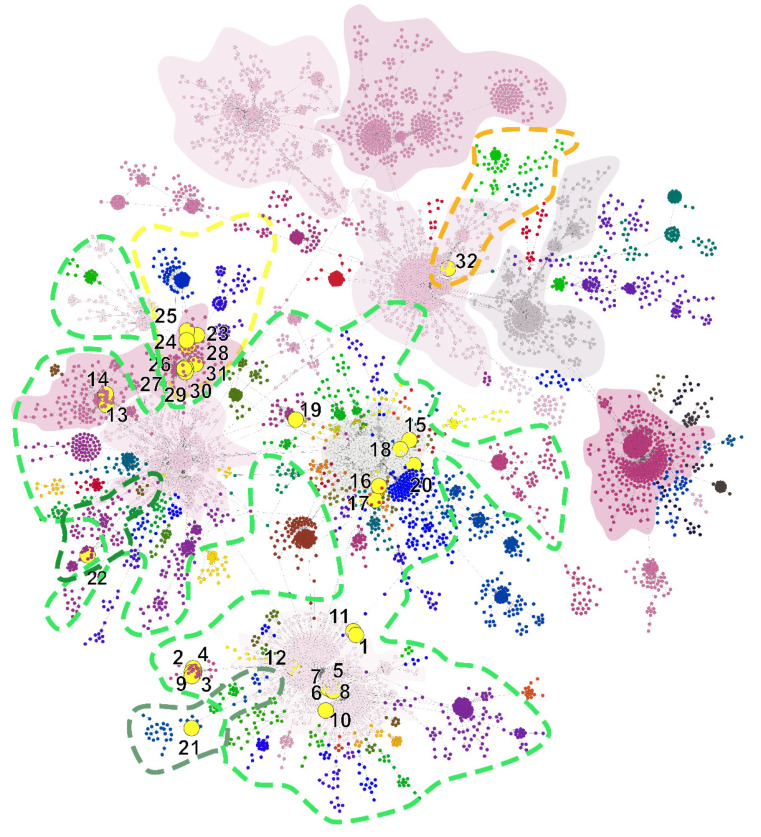
Genetic network analysis of 7864 SARS-CoV-2 complete genomic sequences. Hungarian strains are indicated with numbered yellow dots—numbers referring to Table 1. The nine major clades are represented by a solid color. Genetic lineages are marked with colored dotted lines, where green lines are bordering B 1, B 1.1 and B 1.11; yellow and orange lines mark B 1.5 and B 3, respectively. Dark shaded background areas represent the area of defined clusters.

In order to understand the origin of the first wave of Hungarian COVID-19 epidemic in 2019 and provide baseline data for the evaluation of future epidemic events we applied a Minimum Spanning Tree-based network analysis on complete genomic sequence data of SARS-CoV-2 available in GISAID database [11] current to April 21. MST analysis is a powerful visualization tool to understand epidemiological patterns during an outbreak situation [8]. The network showed negative exponential degree distribution which is common regarding scale-free networks [21]. This characteristic network is typical for epidemics [22]. However, several nodes represented a higher frequency in the lower part of the plot which is the tendency associated with small-world networks [23] (Supplementary Figure S1). Altogether, a total of 147 clusters were identified with a Girvan-Newmann community detection algorithm [24]. In consideration of this approach, a total of nine main clusters were described from the dataset of this time-point, which together serve as the base for the remaining smaller clusters and gives a general picture of the worldwide epidemiological linkage (Figure 1). Although the investigated network contained relatively high number of clusters, its diameter is 25, which infers the farthest distance in the matrix between two sequence is 25 steps, whilst the average path length is 8.91 steps. The high cluster rate was supported by the ratio of these two measures. The proportion of present edges from all possible edges in the network was 0.004 (edge density).

**Figure 2:**
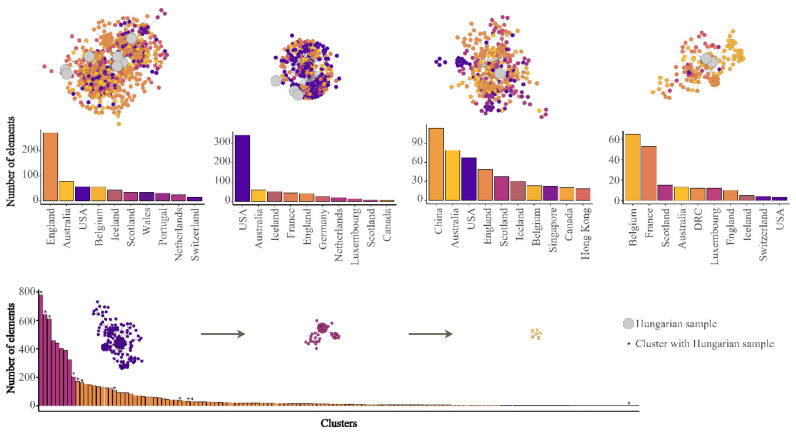
Representation of four main clusters which contain Hungarian SARS-CoV-2 genomes as of 21 April 2020. The ten most common countries of each cluster are summarized in a column graph and represented using different colors. Hungarian sequences are depicted by enlarged grey dots. Number of elements within each remaining (*n* = 147) smaller cluster is indicated as a simple column chart at the bottom of the figure.

The investigated Hungarian genomes are dispersed within four main clusters (Figure 2). The genome designated SARS-CoV-2/human/Hungary/620/27_03_2020 is solely positioned in the B3 genetic lineage, which is a main European lineage with mostly England-related, mainly Welsh sequences [25]. (Table 1; Figure 2, Cluster C). Apart from other Hungarian sequences, this is the only indication for the introduction of B3 lineage into Hungary at the examined time-period in consideration of the available sequence data. All the sequences are dispersed among four main clusters (Figure 2). Two of these are structured by mostly the Western-European sequences, whilst the others are dominant clusters in the USA and the China-Australia-USA relation. Although sampling bias may largely alter the conclusions for the exact geographic origin of a particular strain, the main patterns as multiple introductions from different sources, covering mainly European regions can be concluded based on this dataset.

Using the complete haplotype network dataset as a backbone, we applied additional phylogenetic analysis (Supplementary Figure S2). It is likely that occupation-related movement within the EU resulted in multiple introductory events from Western-European host countries towards Central-Eastern Europe. This observation is further supported by a narrative analysis on the Nextstrain online platform focusing on Eastern European processes of SARS-CoV-2 pandemic evolution [26]. Similarly to Hungary and possibly to the entire region, there were eleven separate introductions to Poland, based on the currently available sequence data [26]. In order to leverage additional support regarding this phenomenon, we applied a local Nextstrain database workflow in the addition of the sequences from this manuscript (Supplementary Figure S3) [27]. As a result of this analysis and considering the observation from Poland, we were able to lend more support for the regular and dispersed introductions into Central-Europe. In addition to regular movement, the border restrictions as outbreak mitigation measures fixed a narrow timescale for individuals returning to Hungary and likely facilitated the parallel introductory events dispersed throughout the country. Based on genetic lineage categorization using PANGOLIN software, 20 out of the total 32 Hungarian sequences fell into the most dominant (i.e., most sequenced) lineage B.1 (Table 1). Dominance may largely depend on sampling heterogeneity between geographic regions and countries. However, it substantiates the connection of Hungary regarding SARS-CoV-2 cases to multiple European sources and provides additional support for the network analysis.

Across the phylogenetic tree (Supplementary Figure S2A), several of the Hungarian sequences were interspersed and mainly clustered with European sequences (England, France, Iceland and Germany) and supported with high posterior probabilities (>80%) while only one Hungarian sequence clustered with a North-American sequence (PP = 95%). These observations elegantly support the scenario regarding multiple individual introductions. In parallel, local clusters were also observed (PP = 100%) indicating local transmission even within the short timeframe of sampling. Moreover, several of the local clusters had very low PP indicating missing data, which is likely to be the consequence of insufficient contact tracing and subsequent missing sequence data.

Within our dataset, the phylogeographic analysis indicated China as the root location (diffusion origin) (Supplementary Figure S2B). Moreover, the virus seemed to spread out to Hungary mainly from Western European countries, nevertheless local transmissions also contributed to disease spread within the country. The data correspond with the epidemiological history of SARS-2-CoV-2 in Hungary [4].

As a support to the phylogenetic conclusions, we present and provide a large-scale haplotype network analysis in reference to the immediate analysis of pandemic evolution of SARS-CoV-2. It is a rapid and useful tool to assess the origin of particular sequences and the acquisition of important data for regarding public health mitigation actions, discovering unidentified infection sources or super-spreading events on a large-scale. In general, it provides the network-based opportunity of rapid, genetic distance-based analysis for all available sequence data, in any context. Herein, we offer this network file available for any researchers to facilitate the understanding of SARS-CoV-2 pandemic evolution. The network file is suitable to visualize any available sequences, available at late April 2020, in its context to all known sequence data.

## 4. Conclusions

The importance of early, country-based mitigation measures are thoroughly exemplified on this dataset. We presented the emergence of multiple virus clusters from various sources in Hungary during the early phase of the epidemic. However, the publicly available epidemiologic data indicate a predominance of confirmed cases in and adjacent to the capital city, Budapest. Possibly, this phenomenon is due to effective mitigation by limiting individual movement, application of social distancing and border restrictions [28]. Therefore, we believe a pan-European, coordinated mitigation policy will be beneficial to prevent significant mixture of European clusters during future epidemics. Here we present the reliability of MST network analysis in genomic epidemiology research. It gives the possibility of powerful visualization and rapid assessment of basic epidemiological patterns, such as source and general transmission patterns of an epidemic.

Our research further highlights the importance of genomic epidemiologic tools for public health decision making. The combination of different methods (i.e., network analysis and phylogenetic approaches) may greatly facilitate the understanding of COVID-19 outbreak evolution.

## Figures and Tables

**Table 1:** Summary of the PANGOLIN software analysis. The table indicates the numbers of Figure 1 and offers additional details for each sample. Background data is also noted where it was available. Letters indicate the Hungarian sequences clusters. Clusters are defined as monophyletic clades with minimum two taxa and at least one Hungarian sequence on Figure S2.

	Taxon Name	Lineage	SH-Alrt	UFbootstrap	Note	Cluster
1	SARS-CoV-2/human/Hungary/49/20_03_2020	B.1	100	100		D
2	SARS-CoV-2/human/Hungary/55/20_03_2020	B.1	100	100		E
3	SARS-CoV-2/human/Hungary/126/22_03_2020	B.1	100	100		E
4	SARS-CoV-2/human/Hungary/186/23_03_2020	B.1	100	100		E
5	SARS-CoV-2/human/Hungary/105w/21_03_2020	B.1	100	100		B
6	SARS-CoV-2/human/Hungary/278w/25_03_2020	B.1	100	100		A
7	SARS-CoV-2/human/Hungary/2801w/25_03_2020	B.1	100	100		A
8	SARS-CoV-2/human/Hungary/3670w/29_03_2020	B.1	100	100		B
9	SARS-CoV-2/human/Hungary/541/27_03_2020	B.1	100	100		E
10	SARS-CoV-2/human/Hungary/777/30_03_2020	B.1	100	100		D
11	SARS-CoV-2/human/Hungary/175/23_03_2020	B.1	100	100		D
12	SARS-CoV-2/human/Hungary/417/25_03_2020	B.1	100	100		E
13	SARS-CoV-2/human/Hungary/3597w/28_03_2020	B.1	100	100		N
14	SARS-CoV-2/human/Hungary/MBL-3/25_03_2020	B.1	100	100	Travel-related: France to Hungary	N
15	SARS-CoV-2/human/Hungary/67/20_03_2020	B.1	100	100		J
16	SARS-CoV-2/human/Hungary/183/23_03_2020	B.1	100	100		K
17	SARS-CoV-2/human/Hungary/419/26_03_2020	B.1	100	100		K
18	SARS-CoV-2/human/Hungary/827/30_03_2020	B.1	100	100		J
19	SARS-CoV-2/human/Hungary/836/30_03_2020	B.1	100	100		M
20	SARS-CoV-2/human/Hungary/792/30_03_2020	B.1	100	100		L
21	SARS-CoV-2/human/Hungary/817/30_03_2020	B.1.1	100	93		C
22	SARS-CoV-2/human/Hungary/572w/29_03_2020	B.1.11	100	99		O
23	SARS-CoV-2/human/Hungary/2/17_03_2020	B.1.5	100	85		G
24	SARS-CoV-2/human/Hungary/MBL-2/23_03_2020	B.1.5	100	74	Household infection	F
25	SARS-CoV-2/human/Hungary/MBL-1/17_03_2020	B.1.5	100	79	Household infection	F
26	SARS-CoV-2/human/Hungary/66/20_03_2020	B.1.5	100	94	Travel-related: Spain to Hungary	G
27	SARS-CoV-2/human/Hungary/1788lc/19_03_2020	B.1.5	100	93		G
28	SARS-CoV-2/human/Hungary/MBL-464/27_03_2020	B.1.5	100	87	Hospital cluster	H
29	SARS-CoV-2/human/Hungary/MBL-465/27_03_2020	B.1.5	100	93	Hospital cluster	H
30	SARS-CoV-2/human/Hungary/MBL-469/27_03_2020	B.1.5	100	93	Hospital cluster	H
31	SARS-CoV-2/human/Hungary/1136/02_04_2020	B.1.5	85	76		I
32	SARS-CoV-2/human/Hungary/620/27_03_2020	B.3	100	87		P

## Supplementary Materials

The following are available online at https://www.mdpi.com/1999-4915/12/12/1401/s1, Supplementary Figure S1: Degree distribution representing the haplotype network analysis; Supplementary Figure S2: Time calibrated phylogenetic and phylogeographic visualization of 105 complete SARS-CoV-2 genomes compared to the 32 Hungarian strains; Supplementary Figure S3: Visualization of Hungarian sequence dataset with Nextstrain local workflow.
