# Supplementary material for: Multiple SARS-CoV-2 Introductions Shaped the Early Outbreak in Central Eastern Europe: Comparing Hungarian Data to a Worldwide Sequence Data-Matrix
Source: Viruses. 2020 Dec 6;12(12):1401. doi: 10.3390/v12121401 (PMC7762115; doi:10.3390/v12121401)
Supplement: Supplementary file 1 [file viruses-12-01401-s001.pdf]

Supplementary Data

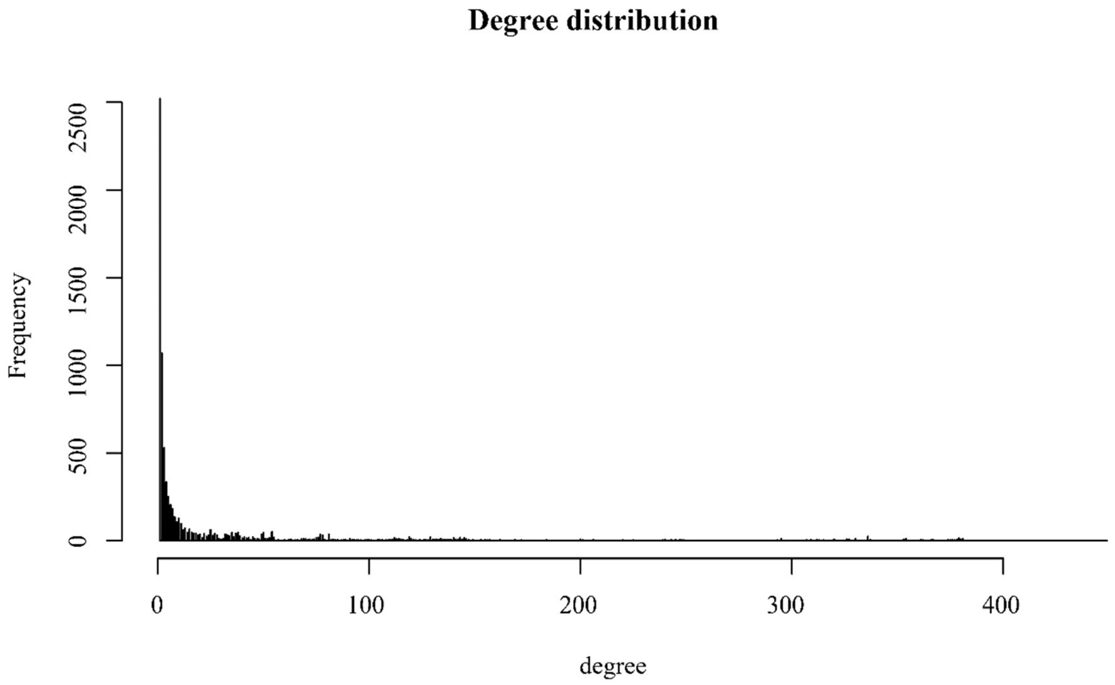

Supplementary Figure 1. | Degree distribution representing the haplotype network analysis

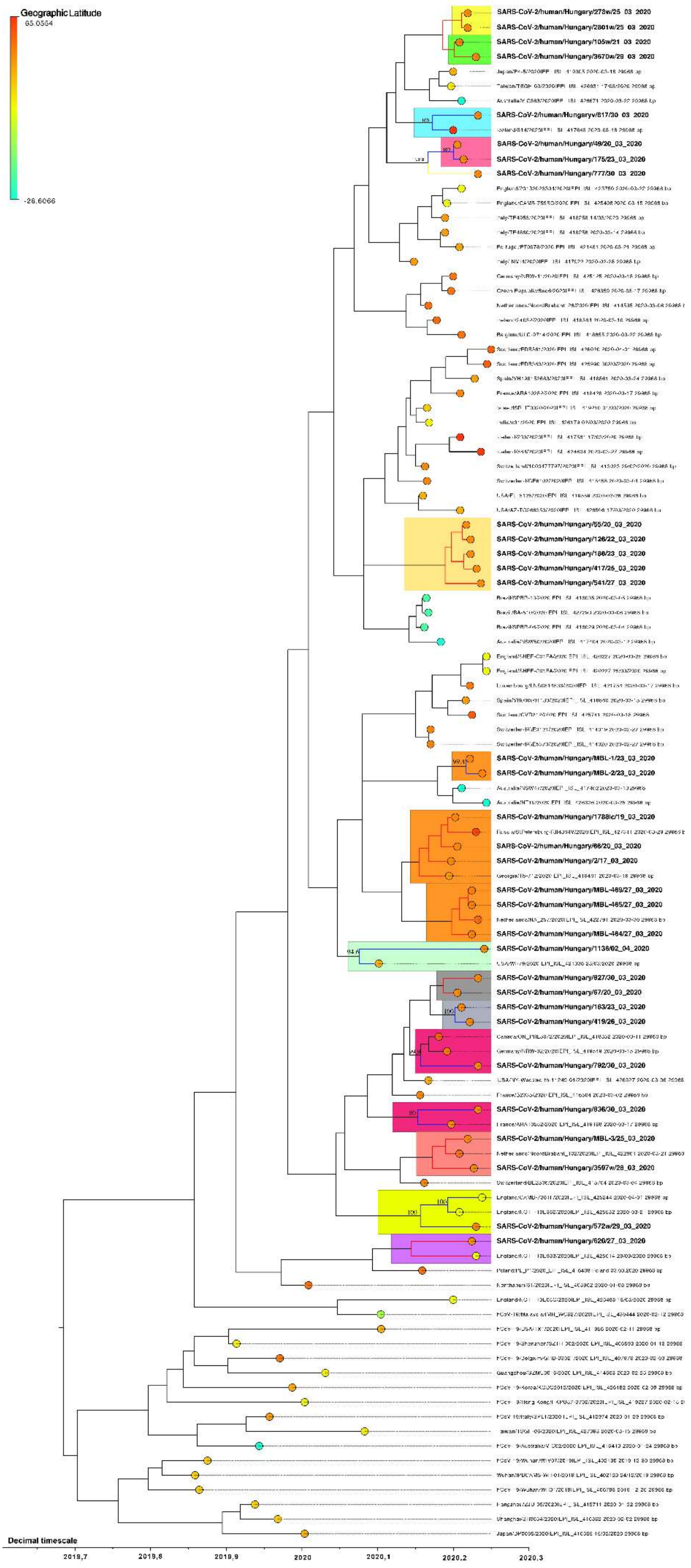

Geographic Latitude
65.0554
-26.6066
SARS-CoV-2/human/Hungary/278w/25_03_2020
SARS-CoV-2/human/Hungary/280w/25_03_2020
SARS-CoV-2/human/Hungary/105w/21_03_2020
SARS-CoV-2/human/Hungary/3570w/28_03_2020
SARS-CoV-2/human/Hungary/49/20_03_2020
SARS-CoV-2/human/Hungary/175/23_03_2020
SARS-CoV-2/human/Hungary/777/30_03_2020
SARS-CoV-2/human/Hungary/55/20_03_2020
SARS-CoV-2/human/Hungary/126/22_03_2020
SARS-CoV-2/human/Hungary/186/23_03_2020
SARS-CoV-2/human/Hungary/417/25_03_2020
SARS-CoV-2/human/Hungary/541/27_03_2020
SARS-CoV-2/human/Hungary/MBL-1/23_03_2020
SARS-CoV-2/human/Hungary/MBL-2/23_03_2020
SARS-CoV-2/human/Hungary/66/20_03_2020
SARS-CoV-2/human/Hungary/2/17_03_2020
SARS-CoV-2/human/Hungary/MBL-466/27_03_2020
SARS-CoV-2/human/Hungary/MBL-465/27_03_2020
SARS-CoV-2/human/Hungary/MBL-464/27_03_2020
SARS-CoV-2/human/Hungary/1138/02_04_2020
SARS-CoV-2/human/Hungary/827/30_03_2020
SARS-CoV-2/human/Hungary/67/20_03_2020
SARS-CoV-2/human/Hungary/163/23_03_2020
SARS-CoV-2/human/Hungary/419/26_03_2020
SARS-CoV-2/human/Hungary/792/30_03_2020
SARS-CoV-2/human/Hungary/836/30_03_2020
SARS-CoV-2/human/Hungary/MBL-3/25_03_2020
SARS-CoV-2/human/Hungary/3597w/28_03_2020
SARS-CoV-2/human/Hungary/572w/29_03_2020
SARS-CoV-2/human/Hungary/626/27_03_2020
100
Decimal timescale
2019,7
2019,8
2019,9
2020
2020,1
2020,2
2020,3

Supplementary Fig. 2 | Time calibrated phylogenetic and phylogeographic visualization of 105 complete SARS-CoV-2 genomes compared to the 32 Hungarian strains. Sequences of this study are highlighted in bold face, colored clades are representing Nextstrain analysis clustering.

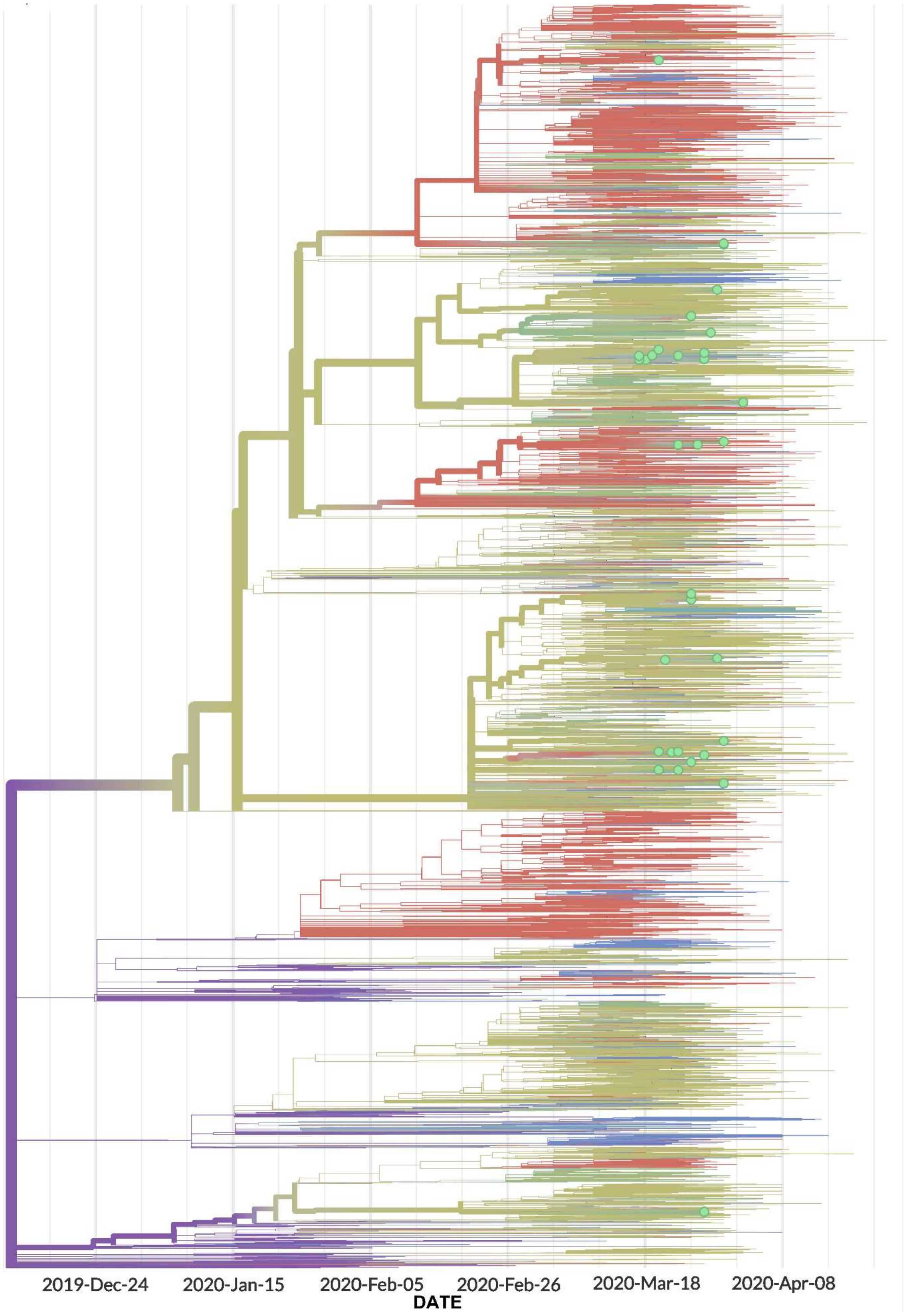

Supplementary Fig. 3 | Visualization of Hungarian sequence dataset with Nextstrain local workflow. Showing 35 (32 from this manuscript and three additional from GISAID database) Hungarian sequences compared to 10,869 genomes sampled between Mar 2020 and Apr 2020.
